# Philosophical ethics and the improvement of farmed animal lives

**DOI:** 10.1093/af/vfz054

**Published:** 2020-01-10

**Authors:** Paul B Thompson

**Affiliations:** Michigan State University, East Lansing, MI

**Keywords:** animal welfare, animal integrity, CAFOs, industrial animal production, quality of life

**Implications**

- Philosophers have neglected the relationships that establish duties to farmed animals, especially in factory farms.
- Many philosophers apparently assume that the conditions in industrial facilities are so horrible that the very idea of discussing obligations to them is vitiated by the unredeemable nature of the circumstances in which they live.
- Even when widely read texts accurately describe welfare deficits, they present a picture which is misleading both as to the extent of these problems, and to difficulty of making changes in response to them.
- Frequently cited welfare problems in factory farms support a case for reform, but it is difficult to see how it would support the claim that what is done to farmed animals is equivalent to torture.

## Introduction

Philosophical reflection on human beings’ morally grounded relationships with nonhuman animals has come a long way since the animal welfare/animal rights debates of the 1970s and 1980s. The basis for granting moral consideration to nonhuman animals expanded far beyond Peter Singer’s discussion of sentience (Singer, 1975) or Tom Regan’s view that human obligation’s toward individuals from other species are grounded in their being “subjects of a life” (Regan, 1983).

Building on the work of Mary Midgley, a number of authors have developed relational theories that interpret human obligations to animals in terms of the specific relationships that we bear to them (Donovan, 1990; MacKinnon, 2004; Anderson, 2004). Clare Palmer’s book *Animal Ethics in Context* articulates an almost fully developed relational ethic in which she explains the basis for differential obligations to wildlife and domesticated animals. In general, humans do not have positive duties to extend care toward wild animals, while bringing an animal into our home establishes duties of care that include attending toward the creature’s biological and emotional needs (Palmer, 2010).

**Figure 1.**
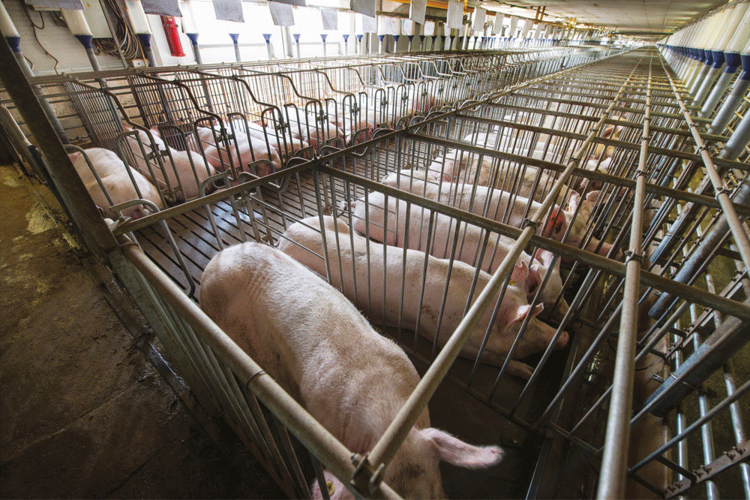
Sows in gestation crates in an indoor swine production system.

With a number of important exceptions, philosophers have neglected the relationships that establish duties to farmed animals. This neglect is especially evident with respect to philosophical studies of livestock being raised in concentrated animal feeding operations (CAFOs), or, colloquially, factory farms (Figure 1).

While, again, there are exceptions, many philosophers apparently assume that the conditions in industrial facilities are so horrible that the very idea of discussing obligations to them is vitiated by the unredeemable nature of the circumstances in which they live. Robert McDowell compares advocacy for amelioration of suffering in CAFOs to claiming that Nazi concentration camps would have been “a lesser evil” if only the prisoners had received a little better treatment prior to being exterminated (Cavell et al., 2008, p. 131). The animal in a factory farm is a cast-off, thoroughly instrumentalized, dominated, abject. The humans who have placed them there cannot fulfill any *moral* obligation to them without relieving them of the torturous conditions in which these animals live. If this is the assumption, however, it is almost never articulated explicitly. Instead, the unspeakable nature of the lives that factory farmed lives is mentioned in passing, as the author moves on to make more philosophically worthwhile points (Thompson, forthcoming).

Are animal ethicists right to view industrially housed animals with such disdain that ethical reflection on their quality of life is simply not worthy of philosophical attention? The argument that they are not correct proceeds in stages. First, as noted, there are philosophers who discuss how CAFOs might be changed to improve animals’ quality of life. A brief survey of some key themes in this literature brings the possibility for an alternative perspective into view. Next, the alternative perspective can be contrasted with claims by well-known animal ethicists. Finally, these better known and highly influential views can be rebutted through a detailed examination of ways that the lives of industrially farmed animals *can* be improved, even if improvement falls short of a full moral justification.

## Philosophy of Improving the Lives of Farmed Animals

It cannot hurt but to begin with the observation that advances since the Singer and Regan era notwithstanding, many if not most academically employed philosophers still seem to fall into one of two camps. Reflection on nonhumans takes one camp all the way to vegetarianism, while those in the other camp have so little interest in animal ethics that they are barely cognizant of the developments in the field. It is, perhaps, natural to conclude that if you are already committed to vegetarianism, there cannot be much reason to think about the condition of animals being raised for food production (Olynk, 2012). Even among the “don’t care” camp, the stridency with which CAFOs are condemned leads many to seek animal products from presumably more humane sources: cage-free eggs, free-range or pasture-raised meats, organic producers, or family farms. Here as with the vegetarians, these mildly proanimal types reach a stopping point. They have no reason to go on and think about the quality of life for animals being raised under the increasingly prevalent industrial conditions. Yet no relations of logical entailment support such inferences. No relational theorist has presented an argument showing that just because you personally have no interest in eating animals, you therefore are discharged of any reason or obligation to consider how their lives might be improved (Thompson, 2015).

In fact, a significant subgroup of philosophers writing in animal ethics do consider the questions that arise in efforts to improve the lives of farmed animals. Contemporaneous with the work of Singer and Regan, Bernard Rollin developed his adaptation of Aristotelean *telos* as a criterion for evaluating the quality of animal lives in a variety of settings, including industrial livestock production. Rollin argued for a view that recognized obligations to individual animals as opposed to maximizing welfare (hence, he has a rights view), but he argues that we derive these obligations from an understanding of an animals’ *telos,* which we understand as the needs and behavioral drives that are typical of creatures from a given species (Rollin, 1991). In later work, it became clear that Rollin views *telos* as determined by an animal’s genetic constitution. While it is wrong to treat animals in ways that fail to respect their *telos,* there is nothing wrong with *changing* an animal’s genetics, whether through breeding or genetic engineering, unless doing so leads to persistent suffering (Rollin, 1995a, 1998).

Rollin also carried out a sustained attack on the industrial conditions under which farmed animals were being raised (Rollin, 1995b). He argued that the growth in CAFOs was the result of a conceptual shift in agricultural and veterinary research. Where researchers had once stressed husbandry, a concept with implicit ethical commitments, they shifted toward animal science, adopting a positivist, value-free attitude toward their research subjects, who were, of course, animals capable of feeling, emotion and better, or worse states of well-being (Rollin, 2004). Rollin’s work became influential in the animal and veterinary sciences, where the lack of fit between an animal’s *telos* and the housing or husbandry began to be used as a guiding approach to applied animal behavior science (Fraser, 1999; Rollin et al., 2012).

Quite a few European philosophers have joined Rollin in helping to determine how the lives of farmed animals might be improved. Peter Sandøe has argued that Singer’s emphasis on sentient experience can be a guide for husbandry (Lassen, Sandøe and Forkman, 2006) and animal breeding (Sandøe et al., 1999). Others have advocated for animal integrity. Like Rollin’s notion of *telos,* animal integrity is intended to convey the sense that each individual animal has status or nature that derives from being the member of a species. It unifies the animal’s cognitive experience of pain or pleasure with more complex relationships that situate the individual within an evolutionary history. Unlike *telos,* integrity is meant to include relationships that derive from the history of domestication by humans and the association with human caregivers, as well as adaptive fit between genetics and the environments in which farmed animals have been bred and raised (Rutgers and Heeger, 1999). Appeals to integrity incorporate considerations that trouble the sanguinity with Rollin regards genetic modification of farmed animals (Bovenkirk, Brom and Van den Bergh, 2002). Bart Gremmen has collaborated with agricultural scientists to apply the notion of animal integrity to determine how applications of precision agriculture could promote animal well-being (Scholten et al., 2013). Mickey Gjerris has also applied the notion in working with co-workers to discuss how industrial livestock facilities may impede the moral perception needed for good husbandry (Harfeld et al., 2016).

This brief survey is far from complete, but it illustrates that different philosophical perspectives on the basis for moral obligations to livestock are being applied in production contexts. Measures are being taken to correct aspects of industrial production systems, even when these measures fall short of abolishing them altogether. Philosophical ethics articulates ways in which the quality of life for livestock in CAFOs can be improved. This work does not imply that improvements justify continued use and development of these systems nor do these authors suggest that with minor changes, factory farming methods will become morally acceptable. What they share is an approach to animal ethics that recognizes the moral significance of contextual elements, including species differences and the peculiarities of production environments. Although the guidance they provide varies, each approach does offer prescriptions that, if acted upon, would make the lives of animals being raised on industrial farms better.

## Mainstream Animal Ethics

Many of the philosophers just discussed work in technical institutes, rather than traditional philosophy departments of prestigious universities. This distinction provides a starting point for the division between work that aims to assist in improving animal lives, and the types of questions taken up in mainstream philosophy. Making improvements in farmed animal quality of life requires the integration of knowledge about animal welfare and knowledge of livestock production systems. Both are value-laden domains, and one role for philosophers has been to articulate and critique value commitments that are implicit within animal welfare science, on the one hand, and philosophies of food production, on the other. All of the above-mentioned philosophers collaborate with researchers who have expertise in applied ethology, veterinary medicine, and animal science. In contrast, research emanating from mainstream philosophy departments is seldom collaborative. The exceptions are collaborations with ethologists working on wildlife, not livestock species (see, e.g., Bekoff and Pierce, 2009).

When mainstream philosophers do mention industrial animal production, the pattern is a sentence or two noting horrific conditions with at most a mention of the general production system, such as caged layers or gestation stalls for pigs. Description of these systems that link their elements to animal welfare deficits are rare. Mainstream philosophers have certainly read Peter Singer’s *Animal Liberation,* which was revised for a 1990 edition and has been updated several other times since its initial publication in 1975. All editions contain a chapter entitle “Down on the Factory Farm” in which Singer describes industrial farming practices that impinge upon an animal’s sentient experience to cause suffering. As with Singer’s collaborations with self-described animal activist Jim Mason (Singer and Mason, 1990, 2007), the chapter works through a series of industrial production systems, beginning with broilers (e.g., chicken meat birds), layers, pigs, veal, dairy, and concluding with beef. In the 1990 edition, this survey is followed by a discussion of slaughter and genetic engineering—two activities that do not necessarily lead to ethical problems, given Singer’s emphasis on pain and suffering. Singer notes that all of these practices are, in principle, amenable to reform, but he also notes that reforms would be unlikely to meet his criterion of equal consideration for human and animal interests (Singer, 2002). (I am working from the 2002 reprint, which contains a new preface where Singer states that the main text is that of the significantly updated 1990 edition. It is worth noting that statements about animal producers’ lack of response to animal welfare critiques that might have been true in 1990 were false in 2002 when Singer’s claims to that effect were reprinted. One can certainly question whether the response was adequate, but by 2002, the European Union had undertaken significant regulatory reform, while North American producers were beginning to make changes through voluntary, cooperative efforts among producers.)

**Figure 2.**
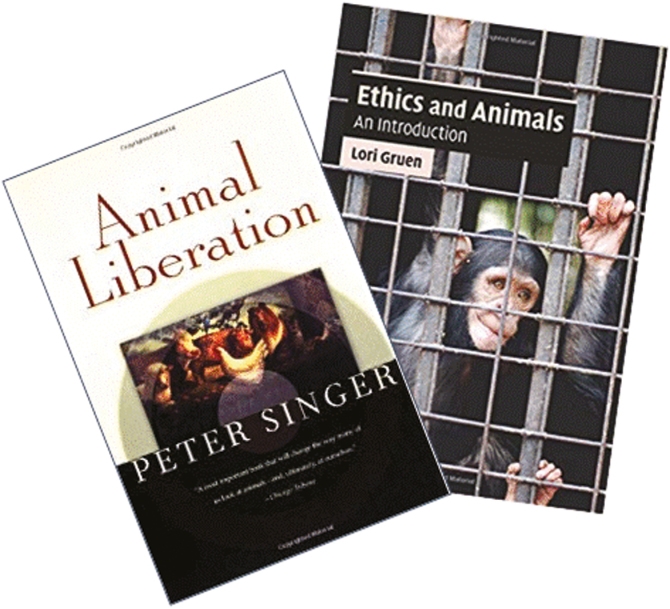
Two books that discuss animal welfare in animal production systems.

Philosophers who collaborate with veterinarians, ethologists, and production specialists would agree that all of the points noted by Singer represent deficiencies in husbandry. There are, however, some points of philosophical difference. The most important difference is more evident in the work of Lori Gruen, who Singer credits as having done research for updates on the farming section of the 1990 edition of *Animal Liberation* (Singer, 2002, p. 313). Gruen’s book *Ethics and Animals: An Introduction* also discusses welfare deficits on industrial farms, though in significantly more inflected language than Singer (Figure 2).

A reader of Gruen’s treatment might easily conclude that *all* animals on factory farms undergo a degree of suffering tantamount to torture at *all* times. Singer is more circumspect in noting that these problems *may* occur, and that they happen on an unspecified but possibly occasional basis. More significantly, Gruen imbeds her discussion of factory farming within an argument for vegetarianism, implying that a vegan diet is the morally appropriate response to the suffering of animal farms (Gruen, 2011). Singer, on the other hand, makes a more qualified and conditional endorsement of vegetarianism (Singer, 2002; Singer and Mason, 2007).

I have no issue with moral vegetarianism or veganism, so long as it is not advanced as a universally applicable norm. The point to emphasize in the present context is that veganism does nothing for the animals that continue to be housed in industrial conditions. What is more, industrial animal production continues to grow on a worldwide basis. The Food and Agriculture Organization (FAO) of the United Nations expects that less industrialized countries will increase production of animal products by 2.5% to 3% annually, and that most of this growth will occur through the importation of industrial production systems (FAO, n.d.). While it may be ethically appropriate for many readers of Singer or Gruen to adopt a vegan diet, it is ethically irresponsible to represent vegetarianism as an ethically adequate answer to the deficits that farmed animals experience in industrial production settings. Yet mainstream philosophy not only seems incapable of making this inference, many mainstream ethicists appear to follow Gruen in alleging that factory farming is utterly irredeemable and incapable of making changes that improve welfare and quality of life.

## Industrially Farmed Animals: A Life Worth Living?

The widely accepted view of industrial animal production may be that of David DeGrazia: “I contend that where the term “factory farming” is properly applied, the conditions of confinement are so intensive that they render the animals’ lives not worth living,” (DeGrazia, 2011, p. 757, italics in the original). If the lives of these animals are worthless, there is nothing to say about how they might be improved. DeGrazia is surely not saying that the lives of farmed animals are unworthy of moral consideration, disposable or literally without value. Nor is it plausible to think that he is implying something like a “wrongful life.” Torts alleging wrongful life involve the claim that only by not having been born could the harms alleged by the plaintiff have been avoided. This condition clearly does not apply to food animals kept in industrial production facilities. DeGrazia implicitly admits that these lives could be improved by removing them from conditions of confinement. Thus, the key point at issue is his claim that conditions on industrial farms render animal lives entirely void of any life-affirming or satisfying experiences.

It is difficult for me to think how I could prove it, but this conclusion seems false to me. I will chip away at the evidence presented in widely read philosophical critiques of industrial animal production, but this is an inductive argument that may not be convincing to all readers. My argument essentially accuses DeGrazia of a hasty generalization. There are, indeed, many issues on factory farm—if there were none, the work I have described to improve conditions there would be pointless. However, a listing of these issues falls short of establishing that all industrially farmed animals endure such extreme suffering as to justify DeGrazia’s gross generalization. In some instances, welfare problems are serious, but limited to a few individuals. In other cases, the conceptual characterization of a welfare deficit is vague or ambiguous in ways that obscure the difference between serious welfare deficits and other conditions that may be less so. In still other cases, welfare deficits may be the lesser of two evils. DeGrazia’s generalization may also fail to acknowledge improvements that have already been made.

The catalog of welfare deficits one finds in Singer or Gruen include many harms experienced by a subset of a flock or herd. A typical scientific study of welfare will report percentage data for welfare deficits observed for the herd or flock. Depending on the production system and breed of animal, these percentages may be comparatively small (e.g., in the range of 2%) or rather large (e.g., 85% or more) (see, e.g., Sherwin, Richards and Nicol, 2010). A 2013 paper on the effects of housing systems (such as concrete flooring) on pigs reports that between 0.1% and 0.05%of sows are removed from production due to lameness annually.

**Figure 3.**
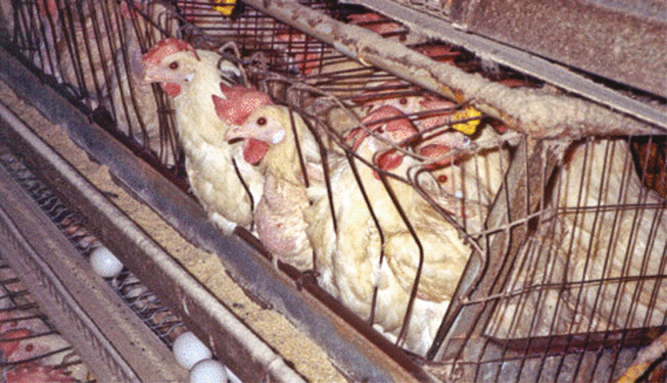
Poultry production in a caged system.

This is enough to be regarded as a serious welfare issue, and the authors of this study compare housing and husbandry approaches with respect to three groups based on visual lameness scores: lame, mildly lame, and nonlame pigs (Grégoire, 2013). Although bone breakage, foot problems, and other painful conditions are documented, one should not conclude that these conditions are universal within CAFOs. Nor should one conclude that the rate of these conditions is necessarily higher in more confined or caged systems: confined laying hens have lower rates of keel bone fractures than uncaged hens (Wilkins et al., 2011; Figure 3).

Stereotypies, or the repetitive, compulsive but apparently nonfunctional movements exhibited by some confined animals are also cited as evidence of a welfare deficit in farmed animals (Singer, 2002, p. 127; Gruen, 2011, p. 85). Defined as a clinical phenomenon, stereotypical behavior is observed in many animal species, including human beings. Two points are relevant to the question of whether stereotypies indicate that an animal’s life is not worth living. First, though certainly observed, dramatic stereotypies such as rapid pacing or persistent pawing are not typical on well-managed farms, industrial or otherwise. Recent work suggests that husbandry may be a more significant cause of welfare problems than the housing system (Hemsworth, 2018). There is thus no reason to think that *all* animals in confined environments are experiencing the overwhelming stress that is often associated with stereotypical behavior. Second, there is a debate over whether stereotypies are reliable indicators of a welfare deficit. In part, this debate reflects differences in the definition of a stereotyped behavior. For example, pigs may exhibit mouthing or chewing, especially prior to feeding times. Although clinical definitions permit classification of these behaviors as stereotypies, researchers currently debate whether they suggest a welfare issue (much less evidence that life is not worth living). Some argue that the evidence on many stereotypies is indicative of a coping behavior that might be observed in virtually any environment. Philosophical ethics could help to clarify disputed issues in this debate (Mason, 2006). In humans, stereotyped behavior is commonly called a *tic*. While tics are indeed characterized as a psychological disorder, they do not necessarily (or even generally) indicate the extreme welfare deficit implied by DeGrazia’s generalization (see Leonard et al., 1992).

Beak trimming is also a frequent target of criticism. Egg producers trim the sharp tip from their hens’ beak in order to limit more serious welfare problems from feather pecking. This comparative normative judgment is an excellent example of where philosophers might weigh in on whether a given practice hurts or helps animal welfare. However, mainstream critics have nothing good to say about beak trimming, which they often refer to as “debeaking” (see Gruen, 2011, p. 83). A review article by Christine Nicol cites literature reporting that properly administered beak trimming does not result in lifelong pain, though botched jobs certainly can and do. She also reports unequivocal findings that given current housing systems, beak trimming reduces mortality (Nicol, 2018). Comparatively, human males are sometimes mutilated shortly after birth by removal of the penal foreskin. When botched, the procedure can lead to lifelong discomfort and stress. Yet one would not conclude that the lives of circumcised men are not worth living.

DeGrazia makes his observation in the context of writing on confinement, so perhaps his remark should be read as laying stress on the crowded and bleak conditions in CAFOs. In fact, crowding is one of the key points where reforms have had some impact over the course of the last 20 years (Scrinis, Parker and Carey, 2017). Significant changes had already been made in the space allotment for U.S. laying hens at the time that DeGrazia wrote (Thompson, 2015). It may be that DeGrazia’s observation is just out of date. Yet, as critics write, hens still have about the space of an 8X10 or A2 piece of typing paper in most production facilities. Increasing space per animal is an entirely legitimate objective for reforming industrial animal production, but the immediate question is whether crowding in industrial farms is so bad that it justifies DeGrazia’s extreme conclusion. Visitors to poultry houses where the sheet of paper stocking density allotment is in effect will, in fact, see quite a bit of open space, as well. Especially when hens are afforded the opportunity for perching (which they are not in classic battery cage systems), they will spend part of their time stretching, dust bathing and wing-flapping, while huddling with other hens at other times (see Keeling and Duncan, 1991; Mench and Duncan, 1998; Dawkins, 2018).

This should not be interpreted to mean that hens do not need more space than they currently have in battery cage systems, especially when stocking rates are based on maximizing return on investment, rather than animal welfare. The observable open space in today’s CAFOs can be an effect normal hen behavior, but it can also be the result of a visitor being there. Behavior when humans are present (especially unknown humans) is quite different than when they are absent, and birds will “flock” or crowd together in response. Singer recounts the behavior of hens during a visit by a reporter as evidence of a welfare deficit (Singer, 2002, p. 114), but it is very likely that the reporter was contributing to the “pandemonium,” simply by being there. On the one hand, this points to yet another epistemological problem for understanding animal welfare: reflexivity. The phenomenon of animal behavior (we should include humans here) is sensitive to the process of being observed. On the other hand, it illustrates how some of the evidence for poor welfare is unfounded.

Physical confinement is unquestionably detrimental to the welfare of sows housed in gestation stalls, another feature of factory livestock farming that is noted by both Singer (Singer, 2002, pp. 126–127) and Gruen (Gruen, 2011, p. 84). Better alternatives are clearly available, yet we should question whether pigs react to this kind of treatment in the way that a human being would. Sows in gestation stalls do not consistently exhibit behavior or physiological indicators of stress, disability, or inability to cope. Researchers have been forced to question the degree to which these types of confinement matter from the perspective of the pig (Hemsworth, 2018). Perhaps these sows are victims of something like the adaptive preference syndrome, where women in abusive relationships with men choose to not only remain, but also defend their abusers (Khader, 2011). That thesis could point toward a philosophical analysis that would aid in reforming pig production. Pigs appear to be more like humans in their capacity for adaptive behavior than any other farmed species, and may have been induced into an apathetic state by their confinement in stalls (Wood-Gush and Vestergaard, 1989). Yet we would neither claim the life of a woman in an abusive relationship is so utterly devoid of satisfaction that it is not worth living, nor that adaptive preferences are sufficient to condemn the entire institution of marriage as incapable of reform.

In summary, this review of frequently cited welfare problems in factory farms does indeed support a case for reform, but it is difficult to see how it would support the claim that what is done to farmed animals is equivalent to torture, as one widely read article in animal ethics put it (Norcross, 2004). Nor is it easy to see why DeGrazia concludes that the lives of these animals are so abject as to be “not worth living.” It is important to stress that my rebuttal to the extremity of DeGrazia’s claim is in no way intended to serve as a justification for current practices on industrial (or any other) animal production facility. Nor do I mean to imply that the quality of life being lived by chickens, pigs, cows, turkeys, or other farmed animals meets a standard of ethical acceptability. Indeed, the larger thrust of this paper is to support the significance of philosophical work that aims to help veterinarians and welfare scientists in their work on improving the conditions in which these animals live. DeGrazia’s statement needs rebutting because it appears to imply that such work is at best an utter waste of time, and at worst supportive of a morally reprehensible institution.

## Conclusion: The Scope of an Engaged Animal Ethics

In summation, I have documented the work of a few philosophers whose work aims to better the conditions on factory farms. This work is not widely appreciated by the philosophical community at large. It is especially shocking how little attention the philosophical community has paid to the work of Bernard Rollin, especially given the influence that he has had beyond academic philosophy. I have elsewhere noted the similarity between Rollin’s work and that of Martha Nussbaum, who apparently has never read him or displays even awareness of his existence (Thompson, 2007). Nussbaum’s emphasis on justice is, indeed, a crucial point not found in Rollin, yet Rollin’s discussion of the link between rights and *telos* would appear to anticipate elements of Nussbaum’s far more celebrated Aristotelean approach.

More seriously, the best known, or as I have called it, mainstream work on animal ethics is entirely dismissive of the potential for reform of industrial animal production. Even when widely read texts accurately describe welfare deficits, they present a picture which is misleading both as to the extent of these problems, and to difficulty of making changes in response to them. I have examined David DeGrazia’s extreme claim that the lives of animals on industrial farms are not worth living as indicative of the unspoken view that appears to be held by most philosophers working in animal ethics. It is a difficult view to rebut. My approach has been to argue that it is an overgeneralization of the conditions that actually exist in CAFOs, and that it oversimplifies the ethical and empirical dimensions of welfare problems as they actually occur. Yet there are many respects in which the work that has been undertaken above is admittedly unambitious.

Doing philosophy to improve the lives of farmed animals is a complicated business, and the above discussion has not really engaged many of the ethical or other questions where philosophical analysis could help. Although I have noted briefly how work by Bernard Rollin, Peter Sandøe, and others is being applied jointly with studies by cognitive ethologists and veterinarians, a reader will need to consult the cited articles to discover *how* their work informs the case for making reforms. My survey of debates over stereotypies, beak trimming and space allotments points indicates that philosophical questions abound in the approaches that are being pursued in response to these problems. Yet I have not specified the nature of these philosophical problems, nor have I developed my own views on them. I have treated all of these topics as beyond the scope of the argument I make in this paper.

**Figure 4.**
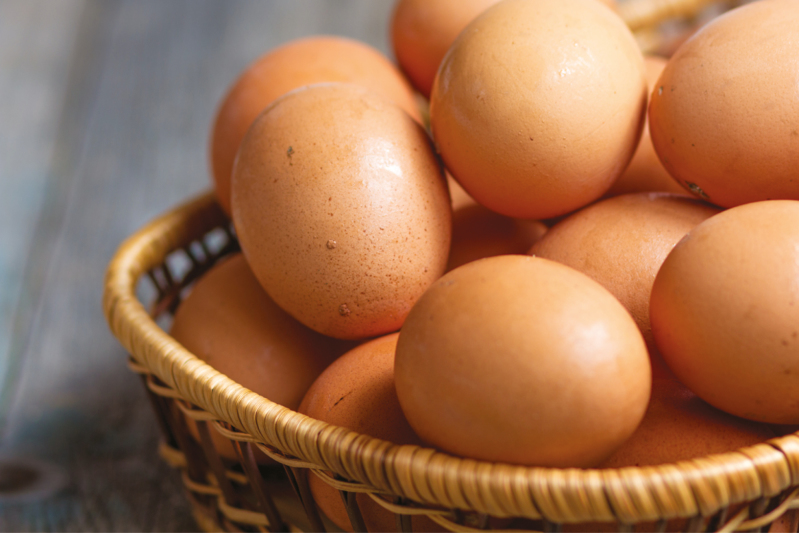
Chicken eggs.

Dimensions of the socio-political environment for reform also pose philosophical issues. As I argue in *From Field to Fork,* the very idea of reforming of industrial production systems implies that there are social constraints on what is possible, and what is ethically desirable. Assumptions must be made to frame these social constraints, and these assumptions are themselves open to philosophical debate. All current models of livestock farming presume that producers must be able to recover their costs through the sale of animal products, for example. This implies that the costs of improving animal welfare must be recouped through the sale of meat, milk, and eggs (Figure 4). The extent to which this can be done depends upon how these markets are structured. For example, direct government intervention seems unlikely in the United States, Canada, or Australia, but the European Union has passed a comprehensive animal welfare law banning certain types of production system (Shields, Shapiro and Rowan, 2017). Even voluntary cooperation among producers to improve welfare was challenged in U.S. courts on the grounds that it is an illegal restraint of trade (Peck, 2015). Classic issues in social and political philosophy permeate the social context in which regulation, cooperation, or competition will impinge upon or promote efforts to improve animal welfare.

There are also large philosophical questions to raise about the various forms of testimony to the conditions in which farmed animals live. I have privileged academic publications, by philosophers and by animal welfare specialists, in the review that I have given here. Yet I would admit that news reports, U-tube videos and social media are more influential in shaping public opinion. To cite only two examples among hundreds, an animal advocacy group released video taken on an Australian egg farm in June of 2019. The video showed workers kicking and pulling the heads of off spent hens, making jokes all the while (Palmer, 2019). In the same month, video of abusive treatment by workers at very large Indiana dairy was released in the United States (McEldowney, 2019). However, another webpage asserts that many abuse videos are staged, and that undercover visitors to factory farms admit privately that they typically find animals in good condition (Peta Kills Animals, n.d.). I do not doubt that at least some of the activist inspired videos and reports capture actual episodes of abuse. Nevertheless, I remain skeptical as to the inference that they prove the extent of abuse apparently presumed by many mainstream philosophers and members of the public, alike. It is also worth mentioning that the abuse shown in the two videos just referenced was all at the hands of human caregivers, and did not ensue as a result of the factory-like conditions in which animals were housed. These videos point, again, to the aforementioned observation that husbandry matters more than the production system (Hemsworth, 2018).

The thrust of this paper has not engaged these critical questions, all of which have a significant philosophical dimension. The animal ethics practiced by philosophers in the most prestigious universities neither addresses these questions nor acknowledges their legitimacy. My goal has been limited: to highlight the extent to which many—arguably most—mainstream philosophers writing on animals have not only failed to address the philosophical issues where analysis could actually help improve the quality of life for animals living on factory farms, they have promulgated philosophical views that actively discourage such inquiry. The lives of animals are worse for this. Surely *that* is an ethical issue in its own right.

## About the Author

**Paul B. Thompson** holds the W.K. Kellogg Chair in Agricultural, Food and Community Ethics at Michigan State University, where he serves on the faculty in the departments of Philosophy, Community Sustainability and Agricultural, Food and Resource Economics. He received his Ph.D. in Philosophy from the State University of New York at Stony Brook and has held posts at Texas A&M University and Purdue University. Thompson’s research and teaching has focused on ethical and philosophical topics in food and agriculture. Thompson has served on advisory boards at the U.S. National Research Council, the U.S. National Academy of Engineering, Genome Canada and for numerous academic journals. His honors include fellowships from the Council on Foreign Relations, the Hastings Center and Yale University, and he is a two-time winner of the Agricultural & Applied Economics Association’s Award for Professional Excellence in Communication.

## References

[CIT0001] Anderson E 2004 Animal rights and the values of nonhuman life. In: Sunstein C. R. and Nussbaum M. C., editors. Animal Rights: Current Debates and New Directions. New York: Oxford University Press; p. 277–298.

[CIT0002] Bekoff M. and Pierce J. 2009 Wild Justice: The Moral Lives of Animals. Chicago (IL): University of Chicago Press.

[CIT0003] Bovenkerk B., Brom F. W. A., and Van den Bergh B. J. 2002 Brave new birds: the use of ‘animal integrity’ in animal ethics. Hastings Cent. Rep. 32(1):16–22. 11917704

[CIT0004] Cavell S., Diamond C., McDowell R. and Hacking I. 2008 Philosophy and Animal Life. New York: Columbia University Press.

[CIT0005] Dawkins M. S 2018 Stocking density: can we judge how much space poultry need? In: Mench J., editor. Advances in Poultry Welfare. Cambridge (MA): Woodhead Publishing; p. 227–242.

[CIT0006] DeGrazia D 2011 The ethics of confining animals from farms to zoos to human homes. In: Beauchamp T. L. and Frey R. G., editors. The Oxford Handbook of Animal Ethics. New York: Oxford University Press; p. 738–768.

[CIT0007] Donovan J 1990 [2007]. Animal rights and feminist theory. In: Donovan J. and Adams C. J., editors. The Feminist Care Tradition in Animal Ethics. New York: Columbia University Press; p. 58–86.

[CIT0008] FAO (n.d.) World Agriculture: Towards 2015–2030. Rome: FAO [accessed September 12, 2019]. Available from http://www.fao.org/3/y4252e/y4252e00.htm#TopOfPage

[CIT0009] Fraser D 1999 Animal ethics and animal welfare science: bridging the two cultures. Appl. Anim. Behav. Sci. 65:171–189.

[CIT0010] Grégoire J., Bergeron R., d’Allaire S., Meunier-Salaün M-C., and Devillers N. 2013 “Assessment of lameness in sows using gait, footprints, postural behaviour and foot lesion analysis.” Animal. 7:1163–1173. 23391233 10.1017/S1751731113000098

[CIT0090] Gruen L 2011 Ethics and Animals: An Introduction. Cambridge (UK): Cambridge University Press.

[CIT0011] Harfeld J. L., Cornoue C., Kornum A. and Gjerris M. 2016 Seeing the animal: on the ethical implications of de-animalization in intensive production systems. J. Agric. Environ. Ethics. 29:407–423.

[CIT0012] Hemsworth P. H 2018 Key determinants of pig welfare: implications of animal management and housing design on livestock welfare. Anim. Prod. Sci. 58:1375–1386.

[CIT0013] Khader S. J., 2011 Adaptive Preferences and Women’s Empowerment. New York: Oxford University Press.

[CIT0014] Keeling L. J., and Duncan I. J. H. 1991 “Social spacing in domestic fowl under seminatural conditions: the effect of behavioural activity and activity transitions.” Appl. Anim. Behav. Sci. 32:205–217.

[CIT0015] Lassen J., Sandøe P., and Forkman B. 2006 “Happy pigs are dirty!–conflicting perspectives on animal welfare.” Livest. Sci. 103(3):221–230.

[CIT0016] Leonard H. L., Lenane M. C., Swedo S. E., Rettew D. C., Gershon E. S., and Rapoport J. L. 1992 Tics and Tourette’s disorder: a 2- to 7-year follow-up of 54 obsessive-compulsive children.” Am. J. Psych. 149:1244–1251. 10.1176/ajp.149.9.1244 1503140

[CIT0017] MacKinnon C 2004 Of mice and men: A feminist fragment on animal rights. In: Sunstein C. R. and Nussbaum M. C., editors. Animal Rights: Current Debates and New Directions. New York: Oxford University Press; p. 263–276.

[CIT0018] Mason G 2006 Stereotypic behaviour in captive animals: fundamentals and implications for welfare and beyond. In: Mason G. and Rushen J., editors. Stereotypic Animal Behaviour: Fundamentals and Applications to Welfare. Cambridge (MA): CABI Press; p. 325–356.

[CIT0019] McEldowney M 2019 Undercover video shows animal abuse at Fair Oaks farm, *Indianapolis Star* June 5 – [accessed September 12, 2019]. Available from https://www.indystar.com/videos/news/2019/06/05/undercover-video-shows-animal-abuse-fair-oaks-farms/1357395001/

[CIT0020] Mench J. A., and Duncan I. J. 1998 Poultry welfare in North America: opportunities and challenges. Poult. Sci. 77:1763–1765. doi:10.1093/ps/77.12.1763 9872576 10.1093/ps/77.12.1763

[CIT0021] Nicol C 2018 “Feather pecking and cannibalism: Can we really stop beak trimming?” In: Mench J., editor. Advances in Poultry Welfare. Cambridge (MA): Woodhead Publishing; p. 175–197.

[CIT0022] Norcross A 2004 Puppies, pigs and people: eating meat and marginal cases. Philos. Perspect. 18:229–245.

[CIT0023] Olynk N. J 2012 Assessing changing consumer preferences for livestock production processes. Anim. Front. 2(3):32–38. doi:10.2527/af.2012-0046

[CIT0024] Palmer C 2010 Animal Ethics in Context. New York: Columbia University Press.

[CIT0025] Palmer E 2019 Farm workers caught on camera pulling chickens’ heads off: ‘Yeah it feels good, look, *Newsweek*, June 16 – [accessed September 12, 2019]. Available from https://www.newsweek.com/animal-liberation-chicken-cruelty-bridgewater-poultry-farm-1444331

[CIT0026] Peck A 2015 “The cost of cutting agricultural output: interpreting the Capper-Volstead Act.” Mo. Law Rev. 80:451–498.

[CIT0027] Peta Kills Animals (n.d.) Fake news – [accessed September 12, 2019]. Available from https://www.petakillsanimals.com/fake-news/

[CIT0028] Regan T 1983 The Case for Animal Rights. Berkeley (CA): University of California Press.

[CIT0029] Rollin B. E 1991 Animal Rights and Human Morality. Buffalo (NY): Prometheus Press.

[CIT0030] Rollin B. E 1995a The Frankenstein Syndrome: Ethical and Social Issues in the Genetic Engineering of Animals. Cambridge (UK): Cambridge University Press.

[CIT0031] Rollin B. E 1995b Farm Animal Welfare: Social Bioethical and Research Issues. Ames: Iowa State University Press.

[CIT0032] Rollin B. E 1998 On *telos* and genetic engineering. In: Holland A. and Johnson A., editors. Animal Biotechnology and Ethics. London: Chapman and Hall; p. 156–171.

[CIT0033] Rollin B. E 2004 Annual meeting keynote address: animal agriculture and the emerging social ethic for animals. J. Anim. Sci. 82:955–964. 15032454 10.2527/2004.823955x

[CIT0034] Rollin B. E., Broom D. M., Fraser D., Golub G. C., Arnot C. and Shapiro P. 2012 Defining agricultural animal welfare: varying viewpoints and approaches. In: Pond W. G., Bazer F. W., and Rollin B. E., editors. Animal Welfare in Animal Agriculture: Husbandry, Stewardship and Sustainability in Animal Production. Boca Raton (FL): CRC Press; p. 75–120.

[CIT0035] Rutgers B, and Heeger R. 1999 “Inherent worth and respect for animal integrity.” In: Dol M., van Vlissingen M. F., Kasanmoentalib S., and Zwart H., editors. Recognizing The Intrinsic Value Of Animals: Beyond Animal Welfare. Assen (NL): Von Gorcum; p. 41–51.

[CIT0036] Sandøe P, Nielsen B. L., Christensen L. G., and Sørensen P. 1999 Staying good while playing God-The ethics of breeding farm animals. Anim. Welf. 8:313–328. 11933931

[CIT0037] Scholten M. C. Th., De Boer I. J. M., Gremmen B., and Lokhorst C. 2013 Livestock farming with care: towards sustainable production of animal-source food. NJAS Wageningen J. Life Sci. 66:3–5.

[CIT0038] Scrinis G., Parker C., and Carey R. 2017 The caged chicken or the free-range egg? The regulatory and market dynamics of layer-hen welfare in the UK, Australia and the USA. J. Agric. Environ. Ethics 30:783–808.

[CIT0039] Sherwin C. M., Richards G. J., and Nicol C. J. 2010 Comparison of the welfare of layer hens in 4 housing systems in the UK. Br. Poult. Sci. 51:488–499. doi:10.1080/00071668.2010.502518 20924842 10.1080/00071668.2010.502518

[CIT0040] Shields S., Shapiro P. and Rowan A. 2017 A decade of progress toward ending the intensive confinement of farm animals in the United States. Animals. 7:40–68. 10.3390/ani7050040 PMC5447922 28505141

[CIT0041] Singer P 1975 Animal Liberation: A New Ethics for Our Treatment of Animals. New York: Random House.

[CIT0042] Singer P 2002 Animal Liberation. New York: HarperCollins.

[CIT0043] Singer P., and Mason J. 1990 Animal Factories. New York: Harmony Books.

[CIT0044] Singer P., and Mason J. 2007 The Ethics of What We Eat. Emmaus (PA): The Rodale Press.

[CIT0045] Thompson P. B 2007 “Ethics on the Frontiers of Livestock Science.” In: Swain D. L., Charmley E., Steel J. W., and Coffey S. G., editors. Redesigning Animal Agriculture: The Challenge of the 21st Century. Wallingrford (UK)/Cambridge (MA): CABI; p. 30–45.

[CIT0046] Thompson P. B 2015 From Field to Fork: Food Ethics for Everyone. New York: Oxford University Press.

[CIT0047] Thompson P. B. (forthcoming). The vanishing ethics of husbandry. In: Bovenkirk B. and Keulartz J., editors. Animals in Our Midst. Dordrecht (NL): Springer.

[CIT0048] Wilkins L. J., McKinstry J. L., Avery N. C., Knowles T. G., Brown S. N., Tarlton J., and Nicol C. J. 2011 Influence of housing system and design on bone strength and keel bone fractures in laying hens. Vet. Rec. 169:414. doi:10.1136/vr.d4831 21862469 10.1136/vr.d4831

[CIT0049] Wood-Gush D. G. M., and Vestergaard K. 1989 Exploratory behavior and the welfare of intensively kept animals. J. Agric. Environ. Ethics. 2:161–169.

